# DUOX2 Expression Is Increased in Barrett Esophagus and Cancerous Tissues of Stomach and Colon

**DOI:** 10.1155/2016/1835684

**Published:** 2015-12-29

**Authors:** Ran Qi, Yunfeng Zhou, Xiaozhen Li, Hong Guo, Lei Gao, Lijuan Wu, Yufeng Wang, Qiang Gao

**Affiliations:** ^1^Department of Gastroenterology and Hepatology, The First Affiliated Hospital, Henan University of Science and Technology, Luoyang, Henan 471000, China; ^2^Shenzhen University Health Science Center, Shenzhen, Guangdong, China

## Abstract

*Aim*. To detect the expression of dual oxidase (DUOX) 2 in Barrett esophagus, gastric cancer, and colorectal cancer (CRC). *Materials and Methods*. The endoscopic biopsies were collected from patients with Barrett esophagus, while the curative resection tissues were obtained from patients with gastric cancer, CRC, or hepatic carcinoma. The DUOX2 protein and mRNA levels were detected with immunohistochemistry (IHC) and real-time quantitative PCR (qPCR). The correlation of DUOX2 expression with clinicopathological parameters of tumors was identified. *Results*. Low levels of DUOX2 mRNA were detected in Barrett esophagus and the adjacent normal tissues, and there was no difference between these two groups. DUOX2 protein was found in Barrett esophagus and undetectable in the normal epithelium. The DUOX2 mRNA and protein levels in the gastric cancer and CRC were increased compared to the adjacent nonmalignant tissues. The elevated DUOX2 in the gastric cancer was significantly associated with smoking history. In CRC tissues, the DUOX2 protein expression level in stages II–IV was significantly higher than that in stage I. In both hepatic carcinoma and the adjacent nonmalignant tissue, the DUOX2 was virtually undetectable. *Conclusion*. DUOX2 in Barrett esophagus, gastric cancer, and CRC may be involved in the tumorigenesis of these tissues.

## 1. Introduction

Reactive oxygen species (ROS) are oxygen-derived small molecules, including hydrogen peroxide (H_2_O_2_), hydroxyl radical, singlet oxygen, and superoxide [1]. ROS serve as intrinsic signaling molecules and regulate normal physiological processes such as cardiac-vascular vessel contraction, immune defense, regulation of transcription, signal transduction, and hormone biosynthesis [2]. When ROS levels are either too low or too high, they cause alteration in cell function and structure. The low ROS levels are related to decreased antimicrobial defense and hypothyroidosis. The abundant ROS may react with proteins, lipids, carbohydrates, and nucleic acids, resulting in cell membrane damage, DNA base modifications, deoxyribose damage, and single- and double-stranded DNA breaks [3]. A large body of evidence shows that excessive ROS are involved in many types of disease process, including inflammation and cancer [4, 5].

There are seven members in the family of ROS-generating nicotinamide adenine dinucleotide phosphate (NADPH) oxidase, including NOX1–5, dual oxidase (DUOX) 1, and DUOX2 [6]. All NADPH oxidases are transmembrane proteins that accept electrons from cytosolic NADPH, transport them through flavin adenine dinucleotide (FAD) and membrane-imbedded hemes, and then donate single electron to reduce oxygen to superoxide [7]. DUOX2 is composed of the NOX-like region at the C-terminal half, two EF hands, a membrane-spanning region, and a peroxidase-like domain at the N-terminus and has an intrinsic Ca^2+^-NADPH-dependent H_2_O_2_-generating activity [8]. The maturation of DUOX2 requires translocation of DUOXs from the endoplasmic reticulum to the apical plasma membrane [9]. DUOX2 has several critical physiological functions, such as thyroid hormone biosynthesis and host defense [10]. DUOX2 is expressed in a variety of tissues, including thyroid gland, airway epithelia, pancreas, and prostate [7, 11]. It was also found in the digestive tract, particularly the distal part [12, 13]. It is believed that DUOX2 in gastrointestinal (GI) tract plays a critical role in host mucosal defense [14–16]. DUOX2 expression can be induced in response to pathologic changes. The microbicidal activity of DUOX2 has been proposed in the DUOX2/LPO/SCN system, in which DUOX2 produced H_2_O_2_ to serve as the substrate for lactoperoxidase (LPO) to produce bactericidal hypothiocyanite by oxidizing the ubiquitous thiocyanate (SCN^−^) in mucosal epithelial cells [17]. DUOX1 and DUOX2 are involved in innate immunity by modulation of mucin secretion, expression of IL-8 and matrix metalloproteinase-9, and executing NOD2-receptor signing in the intestine [17, 18]. Although DUOX2 modulates immune-mediated attack against invading microbial pathogens and actively participates in the signaling pathways against inflammation, overproduction of H_2_O_2_ can lead to oxidative stress resulting in oxidative injuries [7]. In addition to its critical roles in the inflammatory processes, DUOX2 is directly and indirectly involved in the multistage process of carcinogenesis [19, 20].

Recently, much interest and active research efforts have been devoted to ROS, which are implicated in the physiological regulation and pathophysiological processes of many diseases. DUOX2 is a ROS-generating enzyme expressed in the lower gastrointestinal tract. However, the role of DUOX2 in the tumorigenesis of digestive system remains unclear. A few studies showed that DUOX2 was expressed at elevated levels in some human cancers, including the prostate, lung, colon, and breast [7, 19, 20]. However, little is known about the expression of DUOX2 in other regions of human digestive system. In this study, we investigated the levels of DUOX2 expression in precancerous and cancerous digestive tissues, namely, Barrett esophagus, gastric cancer, CRC, and hepatic carcinoma. We will discuss the role of DUOX2 in the pathogenesis of these diseases and its clinical significance.

## 2. Materials and Methods

### 2.1. Patients and Tissues Samples

The endoscopic biopsies from patients with Barrett's esophagus and the curative tumor tissues from patients with gastric, colorectal, and hepatic cancer were collected at the First Affiliated Hospital of Henan University of Science and Technology. The informed consent was obtained from all patients and the study was approved by the clinical research ethics committee of the hospital. The number, gender, and age of patients are listed in Table 1. The control nonlesion tissues were taken at 5 cm away from the lesions in each patient. Barrett esophagus was confirmed by both endoscopy and histology showing that the columnar epithelium replaced normal stratified squamous epithelium [21]. The diagnosis of tumors was done by two experienced pathologists. A section of each sample was fixed in 4% paraformaldehyde, embedded in paraffin, sectioned, and stained with hematoxylin and eosin (HE) staining for pathohistological examination and immunohistochemistry (IHC) detection. The tumor stages were classified according to the seventh edition tumor-node-metastasis (TNM) classification criteria of the American Joint Committee on Cancer [22]. The rest of the sample was stored at −80°C for RNA extraction. RNA* later* (Qiagen, Hilden, Germany, Cat. number 76106) was added immediately after sample collection to prevent RNA degradation.

### 2.2. Quantitative Real-Time PCR (qPCR)

Total RNA was extracted using the TRIzol Reagent solution (Invitrogen, USA) according to the manufacturer's instructions. The cDNA was synthesized from two *μ*g of RNA, using PrimeScript RT Master Mix (Takara, Japan) in a 40 *μ*L reaction mixture, following the procedure: 37°C for 15 min, 85°C for 5 s, and 4°C for 10 min. The primers were designed with the Primer3.0 [23] and synthesized by Sangon Biotechnology (Zhengzhou, China) (Table 2). Real-time qPCR was done with a CFX96 real-time PCR system (Bio-Rad Labs, USA). In 25 *µ*L, the reaction mixture contained 2 *μ*L of cDNA, 12.5 *μ*L of 2 × SYBR Premix Ex Taq II (Takara, Japan), 8.5 *μ*L of H_2_O, and 2 *μ*L of 0.4 *µ*M primers. The PCR reaction consisted of an initial step at 95°C for 30 s, followed by 40 cycles of 95°C for 5 s and 60°C for 30 s. Each sample was assayed in triplicate. The efficiency of PCR amplification was 97% to 104%. A melting curve analysis was performed for the PCR products of target genes to evaluate primer specificity. Relative quantification of target gene mRNA expression was evaluated using the comparative Ct (2^−ΔΔCt^) method and normalized to *β*-actin. The difference of mRNA expression was presented by the relative fold between different groups. When the Ct value is higher than 35, the gene is considered not expressed.

### 2.3. Immunohistochemical (IHC) Staining

Four-micrometer slices were cut from each paraffin-embedded sample and mounted on poly-L-lysine-coated slides. IHC was performed using a modified biotin-peroxidase complex method described previously [24]. Briefly, tissue sections were dewaxed in xylene and rehydrated through graded alcohol. The antigen retrieval was established by microwave boiling of the slides for 10 min in sodium citrate (pH 6.0). Endogenous peroxidase was blocked by incubation for 10 minutes with 3% hydrogen peroxide. After washing, the tissue sections were incubated in goat serum (Sigma, USA) for 20 min and then incubated overnight at 4°C with a rabbit polyclonal antibody specific for DUOX2 (Bioss, Beijing, China, Cat. number bs-11432R). The immunogen for this antibody is KLH-conjugated peptide derived from human DUOX2 (501-600aa). Antigen-antibody detection was performed with a biotinylated-goat-anti-rabbit antibody (Boster Biological Technology Co., Ltd., Wuhan, China, Cat. number SA1020) and subsequently conjugated with horseradish-peroxidase streptavidin. Sections were rinsed and stained with 3,3-diaminobenzidine (DAB) substrate and counterstained with hematoxylin. The thyroid gland tissue was used for DUOX2 positive control; the negative control sections were obtained by omitting the primary antibody or by use of an unrelated rabbit polyclonal antibody.

DUOX2 protein level was assessed with a semiquantitative method. Ten random fields (×400) were selected from each slide and 100 cells per field were counted and scored for the DUOX2 staining. The scores of the staining intensity (none 0; weak 1; moderate 2; strong 3) times the score percentage of positively stained cells (≤5%, 0; 6–25%, 1; 26–50%, 2; 51–75%, 3; >75%, 4) to obtain the semiquantitative scores were as follows: 0 was considered as “−” (negative), <4 as “+”, 4–8 as “++”, and 9–12 as “+++”.

### 2.4. Statistical Analysis

Student's *t*-test, Fisher's exact test, and Mann-Whitney *U* test were used to assess the difference of DUOX2 expression between each pair or subgroups for clinicopathological parameters. One-way ANOVA and a Kruskal-Wallis test were used to compare the data among three or more groups. Significant difference was defined as *P* < 0.05. Data was reported as means ± standard deviation (SD). All statistical analysis was performed by using the SPSS 19.0 statistics package (SPSS Inc., Chicago, IL, USA).

## 3. Results

### 3.1. DUOX2 mRNA Levels Detected by Real-Time qPCR

**Figure 1:**
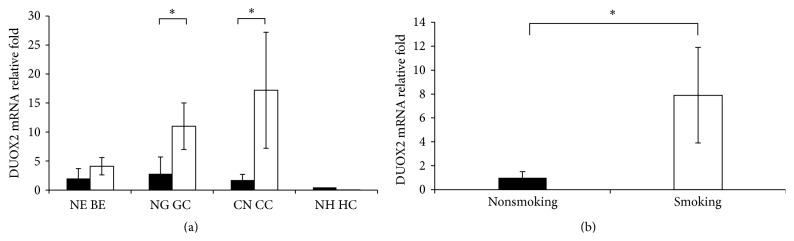
DUOX2 mRNA levels in Barrett esophagus, tumors of stomach, colon, and liver. (a) DUOX2 mRNA was at similar level in normal epithelium of esophagus and Barrett esophagus. The expressions of DUOX2 mRNA in gastric cancer and CRC were both significantly higher than that in each corresponding adjacent nonmalignant tissues. DUOX2 mRNA was hardly detected in hepatic carcinoma and adjacent nonmalignant liver tissue. ^*∗*^ *P* < 0.05. NE: normal esophagus; BE: Barrett esophagus; NG: adjacent nonmalignant gastric tissue; GC: gastric cancer; NC: adjacent nonmalignant colon tissue; CC: CRC; NH adjacent nonmalignant hepatic tissue; HC: hepatic carcinoma. (b) DUOX2 mRNA level in gastric carcinoma of patients with smoking history (*n* = 8) was 7.9-fold higher than that in patients without smoking history (*n* = 16). ^*∗*^ *P* < 0.001.

DUOX2 is a predominant enzyme for the generation of ROS in gastrointestinal tract and involved in mucosal innate immunity and tumorigenesis [19, 25]. Therefore, we measured the DUOX2 expression in normal or nonlesion tissue, precancerous and cancerous tissues in different part of digestive system. Real-time qPCR was carried out to evaluate the DUOX2 mRNA levels in all samples (Figure 1(a)). A low level of DUOX2 mRNA was found in both Barrett esophagus and the adjacent normal esophageal epithelium. There was no difference in DOUX2 mRNA levels between Barrett esophagus and the normal esophagus. Although DUOX2 mRNA was expressed at low levels in adjacent nontumorous tissues, the DOUX2 mRNA in gastric cancer was increased 3.9-fold compared to adjacent nonmalignant control. The DUOX2 mRNA level in the CRC was 10-fold higher than that of nonmalignant tissue. Neither normal nor cancerous liver expressed DUOX2 mRNA.

### 3.2. Detection of DUOX2 Protein Expression by IHC

**Figure 2:**
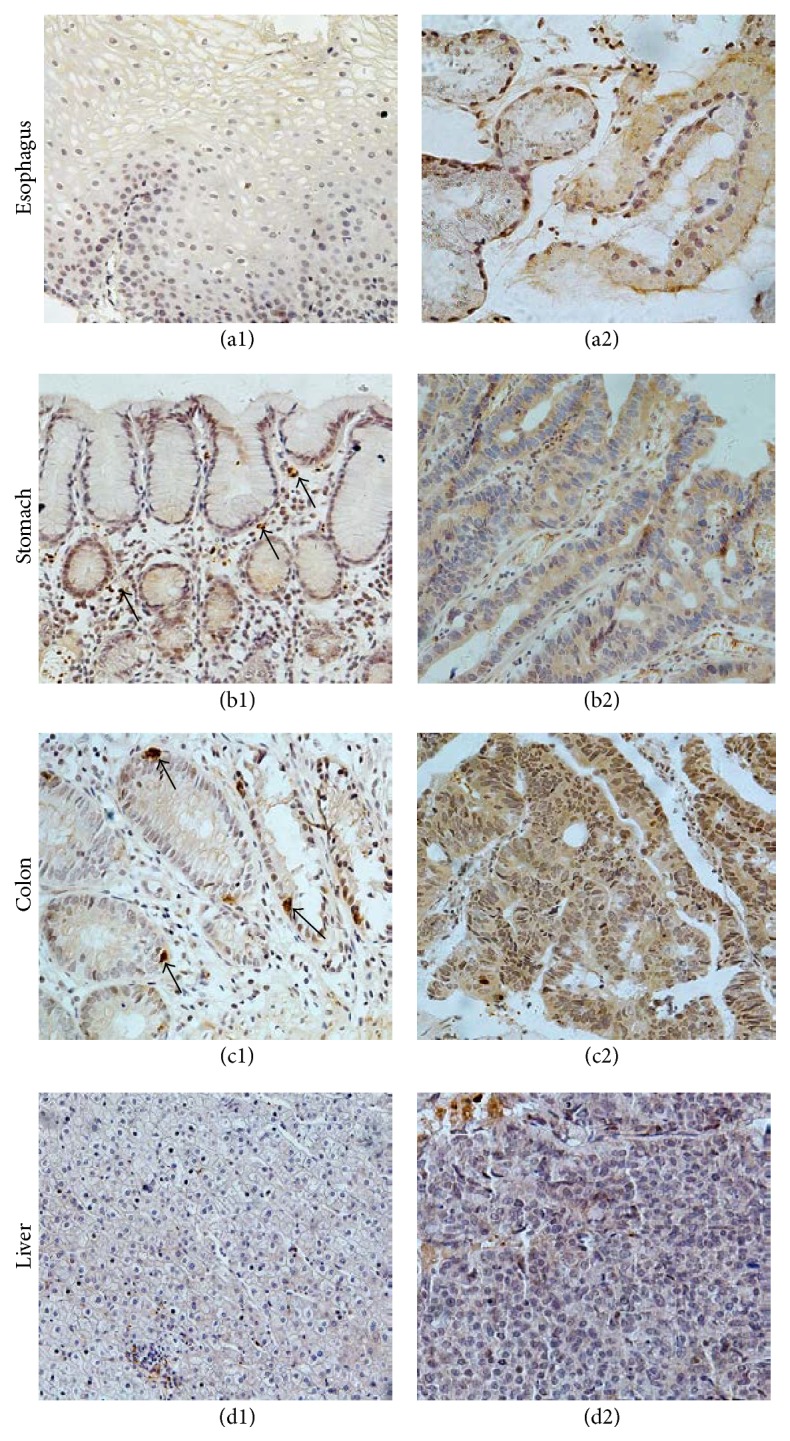
DUOX2 detected by IHC in different tissues of esophagus, stomach, colon, and liver. In normal epithelium of esophagus DUOX2 protein was not detected by IHC (a1); in the columnar epithelium of Barrett esophagus the expression of DUOX2 was mainly located in the apical membrane of epithelial cell (a2); in adjacent nonmalignant gastric tissue epithelial cells were stained negatively with DUOX2 antibody; some unidentified cells were stained positively in lamina propria (b1, arrows); in gastric cancer DUOX2 was mainly found in cell plasma of tumor cells (b2); DUOX2 was at a low level and was mainly at the edge brush of epithelial cells, endocrine cells were strongly stained for DUOX2 (c1, arrows), and DUOX2 expression was higher in CRC; there were nuclei stained with DUOX2 antibody (c2). In hepatic carcinoma and its adjacent nonmalignant tissue there was no DUOX2 IHC staining (d1 and d2). Magnification: ×400.

We further analyzed the expression levels and the location of DUOX2 protein in all biopsies and curative tissues with IHC. The result showed that DUOX2 protein expression was increased in Barrett's esophagus, gastric cancer, and CRC compared to the corresponding nonlesion tissues (Table 3). DUOX2 protein was undetectable in normal stratified squamous epithelium of esophagus (Figure 2(a1)); however, in the columnar epithelium of Barrett esophagus, the DUOX2 protein was detected in the apical membrane of epithelial cell although some epithelial cells have positive stains in the cytoplasm (Figure 2(a2)). Generally the columnar cells expressed a low level of DOUX2 protein.

Similar to DOUX2 mRNA expression, the control nonmalignant gastric tissue had hardly any DUOX2 positive epithelial cells. Curiously, there were some interstitial cells stained positive for DOUX2 in the lamina propria (Figure 2(b1)). Positively stained cells were present in the edge of gastric cancer, while the center of gastric cancer tissue remains negative. The positive staining was mainly found in the cytosol of tumor cells (Figure 2(b2)).

In the adjacent nonmalignant tissue of CRC, a weakly positive DUOX2 staining was detected at the brush edge of epithelial cells. Interestingly, the enteroendocrine cells were strongly stained with anti-DUOX2 antibody (Figure 2(c1)). Furthermore, the CRC tissue had more DUOX2 protein than the control tissue, especially at the circumference of tumor. Consistent with other reports [12, 19], DUOX2 in CRC was evenly located in cytoplasm instead of the apical surface of the epithelial cells. Unexpectedly DUOX2 protein was also detected in the nuclei in tumor cells (Figure 2(c2)).

In the liver, both hepatic carcinoma and adjacent nonmalignant tissue did not have detectable level of DUOX2 protein (Figure 2(d1 and d2)).

### 3.3. Relationship of DUOX2 Expression in Gastrointestinal Cancers with Clinicopathological Features

To further characterize DUOX2, we analyzed the correlations of its expression with clinicopathological parameters of patients with gastric cancer or CRC, including patient gender, age, tumor size, differentiation, lymphatic node metastases, TNM stage, drinking, and smoking history (Tables 3 and 4 and data not shown). The increased expression of DUOX2 mRNA and protein in gastric cancer was significantly associated with smoking history, that is, a higher DUOX2 expression in patients with smoking history (Figure 1(b) and Table 4). Also, DUOX2 protein level in stages II–IV of colorectal cancer was significantly higher than that in stage I (Table 4).

## 4. Discussion

To our knowledge, this is the first report to investigate both DUOX2 protein and gene expression in Barrett esophagus. In this study, a low level of DUOX2 mRNA was found in both normal esophagus and columnar epithelium of Barrett esophagus, although a low level of DUOX2 protein was only observed in Barrett esophagus but not normal esophagus. Barrett esophageal mucosa is gastric-type mucosa with intestinal metaphasia induced by the acid-peptic gastric content, which erodes the squamous mucosa [26]. Barrett esophagus is a well-recognized risk of esophageal adenocarcinoma [27]. Our results show the presence of DUOX2 protein in Barrett esophagus but not in normal esophagus suggesting that DUOX2 may be involved in the carcinogenesis process of Barrett esophagus via ROS production. Others have shown the elevated ROS levels in patients with Barrett esophagus [28]. Thus, DOUX2 may also contribute to esophageal cancer in addition to NOX5, which was shown to promote cell proliferation in esophageal cancer [29].

We also detected that expression of DUOX2 protein and mRNA was significantly increased in gastric cancer. This result is consistent with previous report by Juhasz et al. [20], who also found elevated DUOX2 mRNA expression in gastric tumor compared to adjacent nonmalignant tissue. Chronic inflammation and oxidative stress are important factors for the occurrence of gastric cancer [30, 31].* Helicobacter pylori* (*Hp*) infection is a major risk factor of gastric cancer, and DUOX2 is highly induced in* Hp*-infected rhesus macaques [32]. Increased DUOX2 expression may help to eradicate Hp colonization and infection as suggested by the persistent Helicobacter infection in mice deficient in DUOX2 activity [33]. It is possible that while DUOX2 produced ROS can fend off* Hp* infection, the elevated ROS also injure normal cells and activate procarcinogenic pathways.

Furthermore, DUOX2 mRNA and protein levels were significantly elevated in gastric cancer in patients with smoking history compared to those without smoking history. It was previously reported that smokers had higher levels of DOUX2 expression in the tracheal and airway epithelium than nonsmokers [34]. Smoking is a contributing factor of the carcinogenesis of gastric cancer [31, 35]. Recently it has been reported that smoking suppresses regulatory B cell and inhibits the production of interleukin 10; both events may increase the risk of gastric cancer [36]. Our results suggest that increased DUOX2 expression may contribute to the smoking-induced tumorigenesis of gastric cancer.

Lastly, we showed that CRC tissue had elevated DUOX2 mRNA and protein. This is consistent with Wu et al.'s study [19]. Under physiological conditions, DUOX2 helps host mucosal defense [14–16]. However, overexpression of DUOX2 may lead to excessive ROS production, which can promote tumor progression [37, 38]. A recent study revealed that DUOX2 regulates cell cycle entry via a p53-dependent pathway [39]. Therefore, DUOX2 might participate in the development of GI cancer through ROS-dependent DNA damage and stimulation of cell proliferation. Moreover, the level of DUOX2 protein in tissues of CRC was closely related to clinical tumor stage, indicating that DUOX2 protein could promote tumor metastasis and be used as a predictor for the development and progression of colorectal cancer. The distribution of DUOX2 expression has been reported in colon [12, 13]. However, we found that DUOX2 protein was present in enteroendocrine cells in colon and nuclei staining in CRC cells. The functions of DUOX2 protein in enteroendocrine cells and the nuclei of CRC cells were unclear and worth further study.

## 5. Conclusion

Our study showed that DUOX2 was expressed at a low or undetectable level in the normal control tissues in the digestive system. However, its expression was increased in Barrett esophagus, gastric and colorectal cancer tissues. DUOX2 is H_2_O_2_-generating enzyme and could be a major source of ROS, which have been implicated in the pathogenesis of gastrointestinal disease. Our result suggests that DUOX2 might be involved in the carcinogenesis and development of Barrett esophagus, gastric cancer, and CRC via overproducing ROS, but the exact mechanism was unknown. Further studies are required to clarify the specific mechanism of DUOX2 in tumorigenesis. Better understanding of the role of DUOX2 in digestive system will allow us to evaluate whether DOUX2 can be a novel therapeutic target preventing carcinogenesis in these tissues.

## Figures and Tables

**Table 1:** Patients' clinical features.

Disease	*n*	Gender		Average age
		Male	Female	(Range)
Barrett esophagus	16	11	5	42.4 (31~71)
Gastric cancer	24	20	4	60.8 (38~76)
CRC	39	19	20	63.3 (37~86)
Hepatic carcinoma	6	1	5	65.5 (38~76)

**Table 2:** Primers sequence.

mRNA	Gene	Primer sequence		Product (bp)
NM-014080	DUOX2	Sense	CCTCAGGACCACCATGCTAT	133
		Antisense	TGCAGGGAGTTGAAGAAGG	
NM-001101	*β*-actin	Sense	CTCTTCCAGCCTTCCTTCCT	116
		Antisense	AGCACTGTGTTGGCGTACAG	

**Table 3:** Immunohistochemical staining results.

Disease	Nonlesion tissue	Lesion region	*P*
Barrett esophagus	−	+	0.05
Gastric cancer	−/+	+++	0.01
CRC	+	+++	0.01
Hepatic carcinoma	−	−	—

**Table 4:** The relationship of DUOX2 expressions in gastric cancer and CRC with clinicopathological features.

Characteristics	Protein (*n*)		
	+	++	+++
Gastric cancer			
Smoking history			
No	2	9	5^*∗*^
Yes	0	0	8
CRC			
TNM stage			
I	2	5	0^*∗*^
II–IV	4	8	21

^*∗*^ *P* < 0.05.
